# The effects of different iron and phosphorus treatments on the formation and morphology of iron plaque in rice roots (*Oryza sativa L*)

**DOI:** 10.3389/fpls.2023.1304505

**Published:** 2024-01-08

**Authors:** Haoran Hu, Liyan Bi, Lei Wang, Fangdong Zhan, Xinran Liang, Li Qin, Yuan Li

**Affiliations:** College of Resources and Environment, Yunnan Agricultural University, Kunming, China

**Keywords:** rice root, iron plaque, structure, morphology, Fe^2+^ and PO_4_^3-^

## Abstract

**Introduction:**

Rice (*Oryza sativa L.*) is a pivotal cereal crop worldwide. It relies heavily on the presence of iron plaque on its root surfaces for optimal growth and enhanced stress resistance across diverse environmental conditions.

**Method:**

To study the crystallographic aspects of iron plaque formation on rice roots, the concentrations of Fe^2+^ and PO_4_^3-^ were controlled in this study. The effects of these treatments were assessed through comprehensive analyzes encompassing root growth status, root surface iron concentration, root vitality, enzyme activities, and microstructural characteristics using advanced techniques such as root analysis, scanning electron microscopy (SEM), and ultrathin section transmission electron microscopy (TEM).

**Results:**

The results demonstrated that an increase in the Fe^2+^ concentration or a decrease in the PO_4_^3-^ concentration in the nutrient solution led to improvements in various root growth indicators. There was an elevation in the DCB (dithionite-citrate–bicarbonate) iron content within the roots, enhanced root vitality, and a significant increase in the activities of the superoxide dismutase (SOD), peroxidase (POD) and catalase (CAT) enzymes. Moreover, as the Fe^2+^ concentration increased, amorphous iron oxide minerals on the root surface were gradually transformed into ferrihydrite particles with sizes of approximately 200 nm and goethite particles with sizes of approximately 5 μm. This study showed that an increase in the Fe^2+^ concentration and a decrease in the PO_4_^3-^ concentration led to the formation of substantial iron plaque on the root surfaces. It is noteworthy that there was a distinct gap ranging from 0.5 to 3 μm between the iron plaque formed through PO_4_^3-^ treatment and the cellular layer of the root surface.

**Discussion:**

This study elucidated the impacts of Fe^2+^ and PO_4_^3-^ treatments on the formation, structure, and morphology of the iron plaque while discerning variations in the spatial proximity between the iron plaque and root surface under different treatment conditions.

## 1 Introduction

Rice (*Oryza sativa L*) is a fundamental staple crop for humanity, and it has a profound influence on the survival and progress of human society (Cao et al., 2018). The cultivation of rice holds significant importance as a staple food crop for over three billion individuals globally (Azizi and Lau, 2022). Approximately 90% of the world’s rice is produced in Asia and China is one of the largest global rice producers (Samal et al., 2022). During the growth cycle in soaked soil, rice, like various wetland plants, releases oxygen through roots and oxidizes Fe^2+^ ions near the root surfaces to form iron plaques (Tripathi et al., 2014). The formation of iron plaques enables the absorption of nutrient elements such as phosphorus (P), magnesium (Mg) and zinc (Zn) by the rice (Silva et al., 2023). Iron plaques can also be immobilized by adsorption and coprecipitation of bivalent iron (Fe^2+^), bivalent manganese (Mn^2+^), bivalent cadmium (Cd^2+^) and other heavy metals to reduce their adverse effects on rice (Xu and Yu, 2013).

The iron plaque plays a crucial role in rice growth and has attracted significant attention because it prevents heavy metal poisoning and enhances nutrient absorption. Previous studies indicated that the oxidation capacities of rice roots and the presence of sufficient Fe^2+^ in the soil are crucial prerequisites for the formation of iron plaques. A deficiency of phosphorus in the soil elicits resistance responses in the rice roots, thereby facilitating the formation of iron plaques on the root surface (Deng et al., 2020). Furthermore, implementing a rotational dry and wet field management approach during rice cultivation enhances the root redox potential, root activity and oxygen secretion capacity, thereby facilitating the formation of iron plaque on the roots (Zhang et al., 2019). Huang et al. discovered that the formation of iron plaques on the root surface was promoted by cadmium (Cd) stress. The transporters *OsNRAMP5*, *OsCd1*, *OsIRT1*, *OsIRT2* and *OsYSL15* are involved in the uptake of Fe^2+^ during the process of iron film formation in rice (Huang et al., 2022).

Previous studies were focused on the mechanisms of environmental conditions and rice enzymes, proteins and genes that promoted the formation of root surface iron plaques (Huang et al., 2022). However, limited research has been conducted on the formation and evolution of iron plaques. Iron plaques are fundamentally composed of iron oxides, including ferrihydrite, goethite, hematite and amorphous iron hydroxides (Fu et al., 2016). In non-root soil environments, the transformations of iron oxides and hydroxides are facilitated by structural and morphological changes, which significantly impact cycling of the heavy metal ions (Perez et al., 2021). Torrent et al. discovered that hydropyrite was transformed into goethite in cold regions (Torrent et al., 1982). Grogan et al. observed that ferrihydrite was converted to hematite with enhanced crystallinity under warm conditions. Furthermore, goethite showed the potential to transform into hematite through prolonged aging in the environment (Grogan et al., 2003). Iron oxide minerals with different structures exhibited varying capacities for heavy metal immobilization. Yan et al. demonstrated that the sequential adsorption potentials for Cd decreased in the order ferrihydrite, hematite and goethite (Yan et al., 2021). The morphologies of mineral crystals can determine their capacities for adsorption of heavy metals based on differences in the specific surface area and exposed crystal planes (Li et al., 2022). Ferrihydrite is often found in the form of small particles or gel-like aggregates. Goethite is characterized by a monoclinic crystal system and demonstrates a nanoscale morphology reminiscent of needle-like structures (Michel et al., 2010). Hematite is commonly observed with rhombohedral, plate-like, or circular shapes (Han et al., 2019). The different structures and morphologies of iron plaques formed in various environments influence the capacity of the rice to absorb heavy metals and nutrients. Therefore, the growth of rice is influenced by the structures and morphologies of iron plaques (Weihe et al., 2019).

In soil, the presence of Fe^2+^ and phosphate radical (PO_4_^3-^) affects the structural transformations and morphological evolutions of iron oxide minerals. When Fe^2+^ is present in high concentrations, ferrihydrite undergoes a dissolution-recrystallization process to form goethite (Sheng et al., 2020). Phosphate coats the surface of goethite to impede both structural transformations and morphological evolution processes (Ustunol et al., 2021). Both Fe^2+^ and PO_4_^3-^ play crucial roles in the growth of rice and the formation of iron plaques (Giannopolitis and Ries, 1977; Amako et al., 1994; Sheng et al., 2020; Ustunol et al., 2021). Consequently, this study utilized a hybrid hydroponic system combining sand and liquid for the cultivation of rice plants. This approach, implemented under diverse Fe^2+^ and PO_4_³^-^ treatment conditions, was strategically chosen to reduce the potential interference of soil particles, thereby facilitating more precise observations of iron plaque morphology. Revealing the quantitative aspects of physiological and biochemical parameters in rice root systems. The structures, morphologies and mechanisms of iron plaque formation under different treatments were examined with scanning electron microscopy (SEM) and ultrathin section transmission electron microscopy (TEM). Previous studies focused on the metabolic response of Fe^2+^ and PO_4_^3-^ to the formation of iron plaque on rice root (Fu et al., 2016; Huang et al., 2022). The objective of this study was to explore the effects of Fe^2+^ and PO_4_^3-^ on the microstructure and morphology of iron plaques on rice roots. The results obtained in the present study provide theoretical guidance for the regulation of iron plaques formation in the environment.

## 2 Materials and methods

### 2.1 Rice plant cultivation

The rice variety Tianyou 998 used in this study is more sensitive to Fe^2+^ compared to other rice varieties and forms a larger number of iron plaques (Fu et al., 2012). The rice seeds were cleaned by rinsing with distilled water to remove impurities, and then disinfected with a 10% hydrogen peroxide (H_2_O_2_) solution for 10 minutes. Clean culture dishes were chosen and washed with distilled water. Filter paper was placed at the bottom of each dish, and nutrient solution was added to saturate the filter paper. The sterilized rice seeds were placed on filter paper, and the dishes were incubated at 30 °C for germination.

Healthy and uniform rice seedlings were chosen from the culture dishes. These seedlings were placed in preprepared plastic foam trays and slightly immersed in 1/4 strength complete nutrient solution. The nutrient solution was replaced every 3 d, and the rice plant growth was monitored. When the rice plants reached the three-leaf stage, they were transplanted into PVC pipes containing quartz sand and cultivated for 21 d. The nutrient solution was replaced every 3 d during this period. After 21 d, the rice roots were meticulously extracted and cleaned for subsequent analyzes.

### 2.2 Preparation of complete nutrient solution

The composition of the complete nutrient solution used in this experiment was as follows: NH_4_NO_3_ 0.429 mmol/L, Ca(NO_3_)_2_·4H_2_O 1 mmol/L, MgSO_4_·7H_2_O 1.667 mmol/L, KH_2_PO_4_ 1 mmol/L, K_2_SO_4_ 0.16 mmol/L, Fe(III)-EDTA 50 μmol/L, MnSO_4_ 9.1 μmol/L, ZnSO_4_ 0.15 μmol/L, CuSO_4_ 0.16 μmol/L, (NH_4_)_4_MoO_24_·4H_2_O 0.52 μmol/L, and H_3_BO_3_ 19 μmol/L. The stock solution was prepared with the aforementioned formula (Fu et al., 2016).

### 2.3 Experimental design for rice hydroponic cultivation

To investigate the effects of the Fe^2+^ and PO_4_^3-^ concentrations on the formation of rice arbuscular mycorrhizal iron plaques, an iron-phosphorus ratio experiment was devised. Four different FeCl_2_·4H_2_O concentrations were selected, with three parallel experiments for each concentration: (1) the Fe0.4 treatment: 0.4 mmol/L FeCl_2_·4H_2_O, (2) the Fe1.2 treatment: 1.2 mmol/L FeCl_2_·4H_2_O, (3) the Fe2.0 treatment: 2.0 mmol/L FeCl_2_·4H_2_O, and (4) the Fe3.2 treatment: 3.2 mmol/L FeCl_2_·4H_2_O. All rice experiments were carried out with a phosphorus concentration of 0.04 mmol/L. The rice plants were cultured for 2 d, after which varying iron concentrations were introduced while maintaining a constant phosphorus concentration.

Similarly, to explore the effects of PO_4_^3-^ concentrations on the formation of rice arbuscular mycorrhizal iron plaques, another iron-phosphorus ratio experiment was conducted. These experiments included four PO_4_^3-^ concentrations: (1) the P0.1 treatment: 0.1 mmol/L KH_2_PO_4_, (2) the P0.033 treatment: 0.033 mmol/L KH_2_PO_4_, (3) the P0.02 treatment: 0.02 mmol/L KH_2_PO_4_, and (4) the P0.01 treatment: 0.01 mmol/L KH_2_PO_4_. As with the Fe experiments, after 2 d of cultivation with the corresponding KH_2_PO_4_ concentration, the same iron concentration (0.1 mmol/L Fe) was introduced while keeping the phosphorus concentration constant. The rice plants were cultured for an additional 2 d.

### 2.4 Measurement of rice arbuscular root system and dithionite-citrate—bicarbonate (DCB)-Fe on rice arbuscular root surface

Tweezers are used to pick out the quartz sand one by one to delicately remove the rice roots. The rice roots were delicately removed, washed with distilled water, placed on a flat surface, and scanned with a root scanner to record the data.

Standard solutions with known DCB-Fe concentrations were prepared. These solutions had concentrations of 1 μg/mL, 2 μg/mL, 3 μg/mL, 4 μg/mL, and 5 μg/mL. The absorbance values of these solutions were utilized to generate a standard curve.

### 2.5 Root vitality measurement

After 24 h of treatment, the fresh rice roots (0.2 g) were extracted for enzyme measurements. The superoxide dismutase (SOD) activity was measured with the method of Jiang et al., reported (Jiang et al., 2023), and one unit of activity inhibited the reduction of nitroblue tetrazolium (NBT) by 50%. The peroxidase (POD) and catalase (CAT) activities were determined with the guaiacol method and potassium permanganate titration method, respectively (Thounaojam et al., 2012).

### 2.6 Measurement of root growth and vitality

The main root length, total root length, root surface area, and root volume were measured with a root scanner. The rice root vitality was assessed with the amount of α-naphthylamine oxidation over 24 h (Zhang et al., 2021).

### 2.7 Scanning electron microscopy (SEM) measurement of rice arbuscular root iron plaques

The rice roots, labeled according to their concentrations, were washed with deionized water. Subsequently, they were dried at 75°C for 4 h in an oven and analyzed with a field emission scanning electron microscope (FESEM) (Hitachi SU801, Japan). A small amount of powder was placed on conductive tape, and the sample surface was observed after platinum/carbon coating. The acceleration voltage was 5 kV, the current was 5 μA, and the working distance (WD) was 7.9 nm.

### 2.8 Transmission electron microscopy (TEM) analysis of rice arbuscular root iron plaques

Deionized water-washed rice roots were carefully placed into 1.5 mL conical tubes. Subsequently, 0.5 mL of a fixative solution, which contained a 2.5% glutaraldehyde solution, was added to each tube. These tubes were then subjected to centrifugation for 1 hour to create compact aggregates. Following centrifugation, the fixative solution was carefully poured into the centrifuge tubes, ensuring complete submersion of the samples. The prepared samples were subsequently cold-fixed at a temperature of 4°C within a refrigerator for a duration of 12 h.

For subsequent stages of processing, the fixed samples were embedded in epoxy resin. To maintain a low temperature during this process, the samples were initially placed in foam boxes accompanied by ice packs. To facilitate the observation process, the prepared samples were sectioned. For this purpose, the samples were embedded in epoxy resin and allowed to polymerize in darkness for a period of 48 h. Subsequently, sectioning was executed with an ultramicrotome equipped with a diamond knife (Leica EM·UC6). The resulting sections, with approximate thicknesses of 50 nm, were then positioned onto porous carbon-coated copper grids for observation.

### 2.9 Statistical analysis

The data were subjected to analysis of variance, and *post hoc* comparisons were carried out with Duncan’s multiple range test at P<0.05. The statistical software program SPSS version 13.0 (SPSS Inc., Chicago, IL, USA) was used.

## 3 Results

### 3.1 Effects of different Fe^2+^ and PO_4_^3-^ concentrations on the root growth of rice

To learn how Fe^2+^ and PO_4_^3-^ affected rice root growth and hence the formation of iron plaques on the root surfaces, different Fe^2+^ and PO_4_^3-^ concentrations were applied during rice growth. The results demonstrated that the primary root length of rice increased from 10.80±0.12 to 12.52±0.30 cm, total root length increased from 126.51±2.69 to 136.77±1.46 cm/plant, root surface area increased from 14.19±0.11 to 18.20±0.16 cm^2^/plant, and root volume increased from 0.175±0.003 to 0.192 ±0.002 cm^3^/plant within the Fe^2+^ concentration range of 0.4 to 3.2 mmol/L (**Table 1**). We found that the taproot, total root, root surface area and root volume of rice exhibited increasing trends as the concentration of Fe^2+^ in the nutrient solution was increased from 0.4 to 3.2 mmol/L. This suggested that the presence of Fe^2+^ in the environment promoted rice root growth. The taproot length, total root length, and root surface area of the rice exhibited significant increases when the concentration of PO_4_^3-^ in the nutrient solution was raised from 0.01 to 0.1 mmol/L. The increases in the fresh root weight and the root volume, however, were not pronounced, possibly due to the reductions in root diameter with PO_4_^3-^ deficiencies. This was consistent with previous findings indicating that a PO_4_^3-^ deficiency promoted plant root growth, increases in the root hair length and/or density and a decrease in the root diameter, ultimately resulting in a greater surface area for soil probing (Gahoonia et al., 2001; Deng et al., 2020).

**Table 1:** Root growth of rice under different treatments.

Treatment group	Primary root length (cm)	Total root length (cm/plant)	Root surface area (cm^2^/plant)	Root volume (cm^3^/plant)	Fresh root weight (g/plant)
Fe0.4	10.80±0.12 c	126.51±2.69 c	14.19±0.11 c	0.175±0.003 b	0.048±0.003 c
Fe1.2	11.63±0.07 b	129.45±1.83 c	14.47±0.16 c	0.182±0.003 ab	0.052±0.001 bc
Fe2.0	12.36±0.08 a	130.7±1.53 ab	16.18±0.13 b	0.187±0.005 a	0.057±0.004 b
Fe3.2	12.52±0.30 a	136.77±1.46 a	18.20±0.16 a	0.192±0.002 a	0.065±0.002 a
P0.01	7.61±0.20 d	115.56±1.12 c	11.41±0.97 d	0.031±0.004 d	0.027±0.001 c
P0.02	8.66±0.12 c	120.10±3.15 bc	12.68±0.82 c	0.038±0.002 c	0.034±0.003 c
P0.033	10.28±0.03 b	125.04±3.18 b	14.55±0.69 b	0.045±0.003 b	0.043±0.003 b
P0.1	13.57±0.05 a	135.56±2.42 a	17.54±0.57 a	0.057±0.004 a	0.054±0.001 a

Values followed by different letters in a column are significant differences among cultivars at the 5% level.

### 3.2 Effects of different Fe^2+^ and PO_4_^3-^ concentrations on iron plaques of rice roots

**Figure 1:**
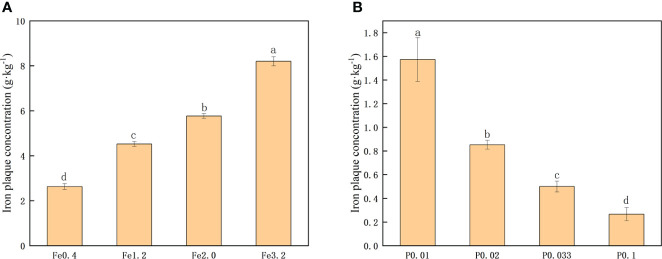
**(A)** Iron plaque concentration under different Fe^2+^ treatments; **(B)** Iron plaque concentration under different PO_4_^3-^ treatments. Different letters indicate significant differences among treatments at p < 0.05 level.

The extraction of iron from iron plaques on the rice root surface of DCB was employed to quantitatively compare the extent of iron plaque formation (Zhang et al., 2023). As depicted in **Figure 1**, an increase in the concentration of extrinsic Fe^2+^ in the nutrient solution resulted in a significant elevation of the extracted DCB-Fe concentration (P>0.05). These findings suggested that augmenting the Fe^2+^ concentration promoted iron plaque formation on the root surface. When controlling the Fe^2+^ concentration at 0.3 mmol/L, only the PO_4_^3-^ concentration in the nutrient solution was changed. **Figure 1B** shows that an increase in the PO_4_^3-^ concentration resulted in a decrease in the DCB-Fe concentration on the root surface. The increased PO_4_^3-^ concentration in the nutrient solution significantly hampered the formation of iron plaques on the root surfaces.

### 3.3 Effects of different treatments on the activities of antioxidant enzymes

**Figure 2:**
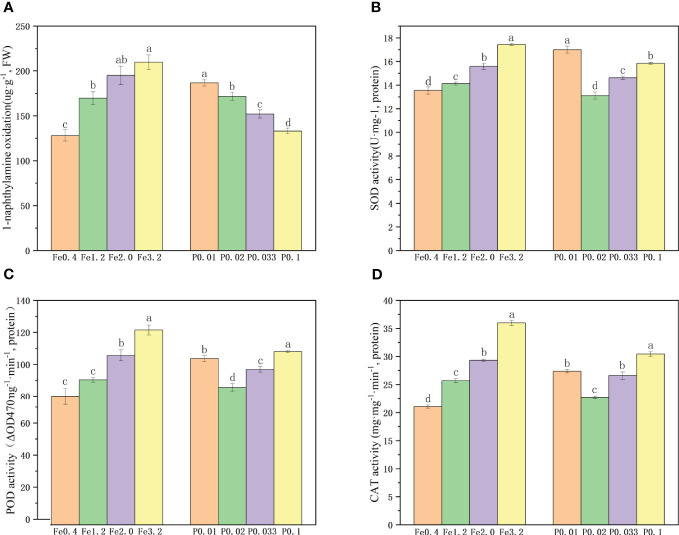
**(A)** Root activity under different Fe^2+^ and PO_4_^3-^treatments; **(B-D)**. Antioxidant enzyme activities of roots under different Fe^2+^ and PO_4_^3-^ treatments; Different letters indicate significant differences among treatments at p < 0.05 level.

The vitality of the rice root was evaluated based on the oxidation of α-naphthylamine with varying concentrations of Fe^2+^ and PO_4_^3-^. The vitality of the rice roots gradually improved with increasing concentrations of Fe^2+^ in the nutrient solution (**Figure 2A**). When the Fe^2+^ concentration reached 3.2 mmol/L, there was only a slight enhancement in the rice root vitality, suggesting that a higher concentration of Fe^2+^ did not exert a significant promotive effect on vitality. Cultivating the rice roots in a PO_4_^3–^enriched nutrient solution provided an initial increase in the root vitality with increasing PO_4_^3-^ concentration. The PO_4_^3-^ deficiency negatively impacted the root vitality of the rice.

We quantified the activities of the root SOD, POD and CAT enzymes in rice roots exposed to varying concentrations of Fe^2+^ and PO_4_^3-^. The findings demonstrated a 36.7 % increase in the SOD enzyme activity, a 32.4% increase in the POD enzyme activity, and a 33.6% increase in the CAT enzyme activity with increases in the Fe^2+^ concentration from 0.4 mmol/L to 3.2 mmol/L, indicating that higher Fe^2+^ levels promoted root vitality and enhanced enzymatic function (**Figures 2B-D**). When the concentration of PO_4_^3-^ was increased from 0.01 mmol/L to 0.02 mmol/L, the activities of the SOD, POD and CAT enzymes decreased. However, the activities of these three enzymes exhibited increases with further increases in the concentration of PO_4_^3-^ from 0.02 mmol/L to 0.1 mmol/L (**Figures 2B-D**), suggesting that both moderate increases in PO_4_^3-^ concentration and its deficiency can positively impact root vitality and enzyme activity.

### 3.4 Effects of different treatments on the morphology of iron plaques

**Figure 3:**
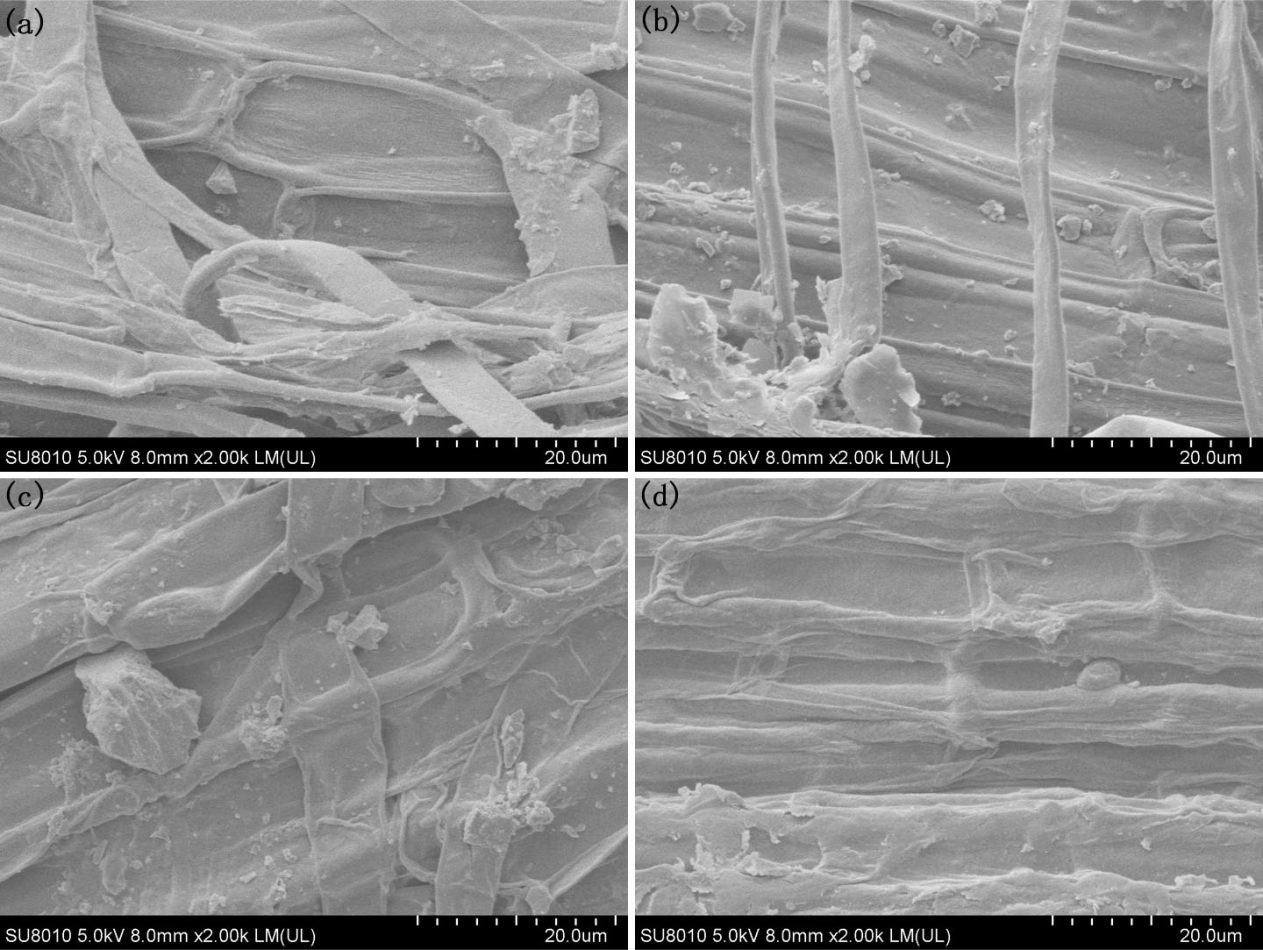
SEM morphology of the root under different Fe^2+^ treatments, **(A)** Fe0.4; **(B)** Fe1.2; **(C)** Fe2.0; **(D)** Fe3.2.

To investigate the morphologies of rice iron plaques treated with different Fe^2+^ and PO_4_^3-^ nutrient solutions, the rice roots were dried and observed with SEM. The results revealed that upon treatment with an Fe^2+^ concentration of 0.4 mmol/L, the rice root surfaces exhibited a distribution of small iron oxide particles measuring approximately 1 μm, along with larger particles measuring approximately 6 μm when observed at a magnification of 2000 times (**Figure 3A**; **Supplementary Figure 1A**). When the concentration of Fe^2+^ was increased to 1.2 mmol/L, a significant increase in the number of particles on the surfaces of the rice roots was observed, as shown in **Figure 3B**; **Supplementary Figure 1B**. Moreover, these particles exhibited a predominant morphology with larger particles measuring approximately 2 μm, along with larger iron oxide particles measuring approximately 10 μm. As the concentration of Fe^2+^ was increased to 2.0 mmol/L, distinct particles were observed on the rice root surfaces, with sizes of 13 μm (large), 5 μm (medium) and less than 1 μm (small) (**Figure 3C**; **Supplementary Figure 1C**). This observation suggested that the elevated Fe^2+^ levels facilitated the dissolution and subsequent recrystallization processes of the iron plaque particles present on the root surfaces. The concentration of Fe^2+^ was raised to 3.2 mmol/L, and the particles on the root surfaces vanished to give a homogeneous iron plaque. The surface was uniformly covered by an iron plaque with a thickness of approximately 1 μm (**Figure 3D**; **Supplementary Figure 1D**).

**Figure 4:**
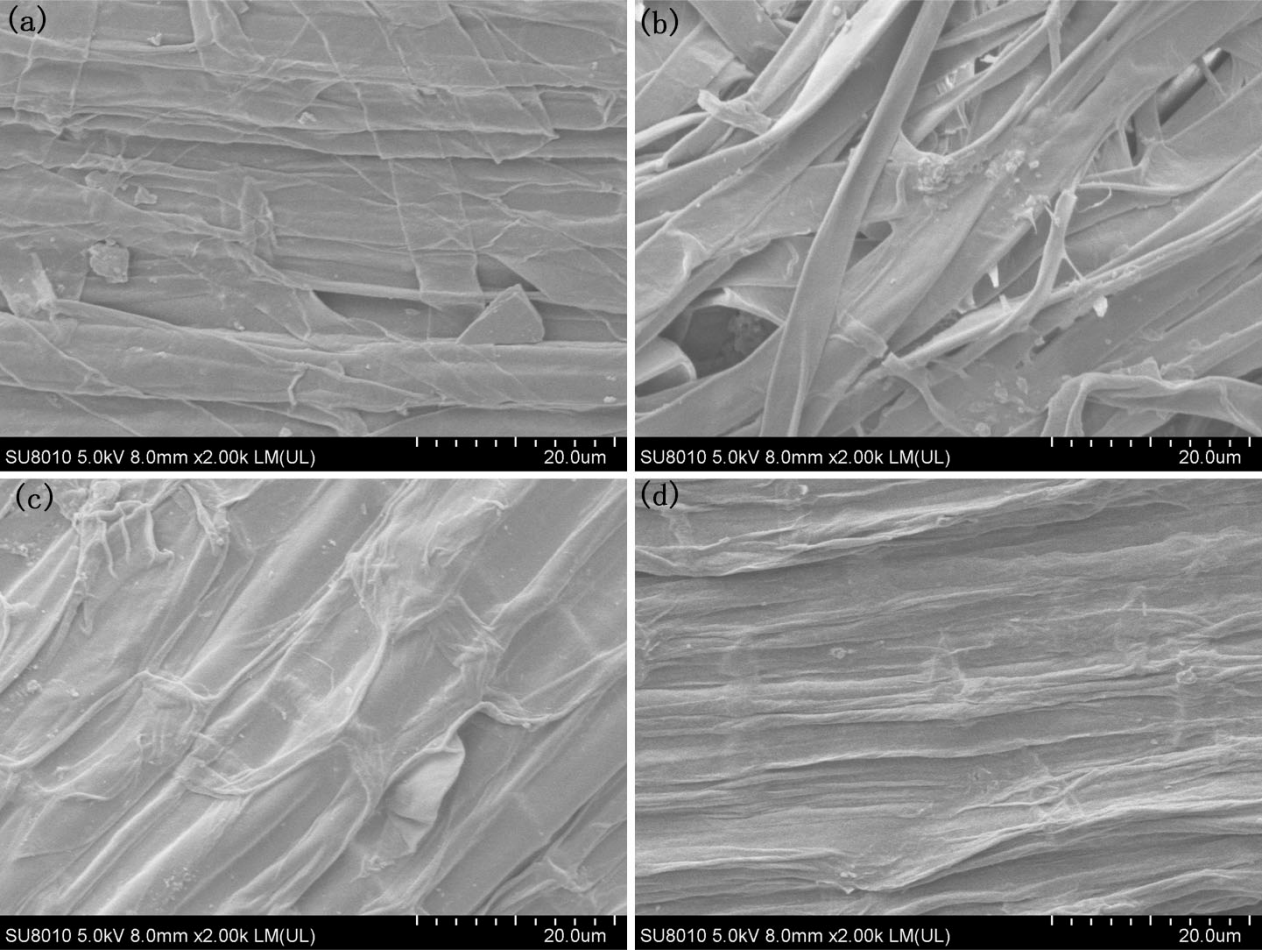
SEM morphology of the root under different PO_4_^3-^ treatments, **(A)** P0.01; **(B)** P0.02; **(C)** P0.033; **(D)** P0.1.

The morphological changes occurring during the formation of iron plaque on the rice root surface when using a PO_4_^3-^ nutrient solution exhibited an opposite trend to that observed with Fe^2+^ treatment. Following cultivation with 0.01 mmol/L PO_4_^3-^, there was a relatively low occurrence of larger particles measuring 3 μm on the surfaces of the rice roots (**Figure 4A**; **Supplementary Figure 2A**). The particle sizes on the rice root surfaces decreased to less than 1 μm as the concentration of PO_4_^3-^ was increased to 0.033 mmol/L, and the quantity decreased (**Figures 4B, C**; **Supplementary Figure 2C**). When the concentration of PO_4_^3-^ reached 0.1 mmol/L, the rice root surfaces exhibited smooth textures devoid of any discernible particles (**Figure 4D**; **Supplementary Figure 2D**), implying a gradual reduction and eventual disappearance of the iron oxide particles with increasing PO_4_^3-^.

**Figure 5:**
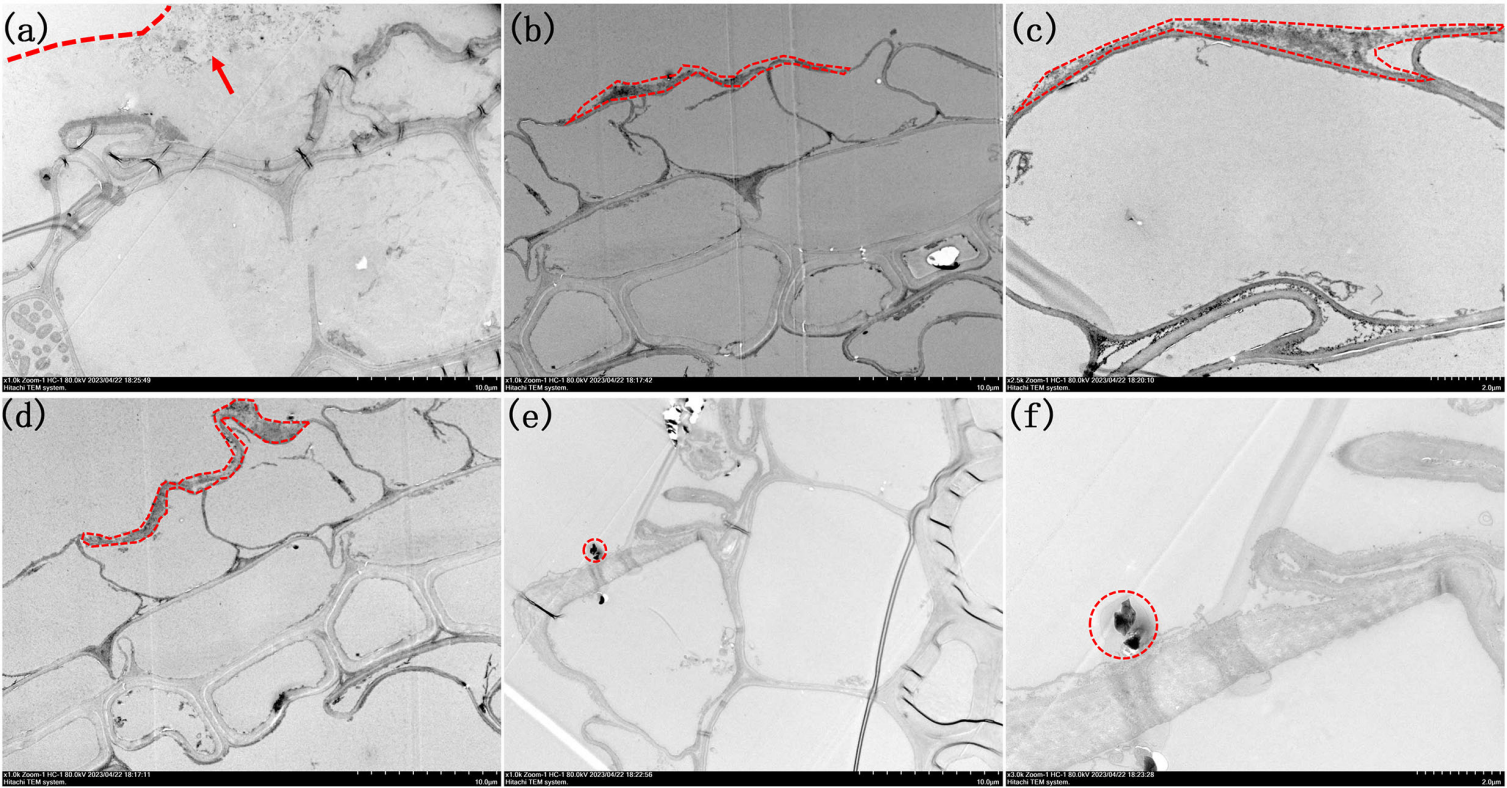
Morphologies of root TEM sections under different Fe^2+^ treatments. **(A)** 1000x by Fe0.4; **(B)** 1000x of Fe1.2; **(C)** 2500x of Fe1.2; **(D)** 1000x of Fe2.0; **(E)** 1000x Fe3.2; **(F)** 3000x of Fe3.2. Arrow in image **(A)** highlight the presence of small nanoparticles inside the colloidal film. The iron plaques are outlined in **(A-F)**.

**Figure 6:**
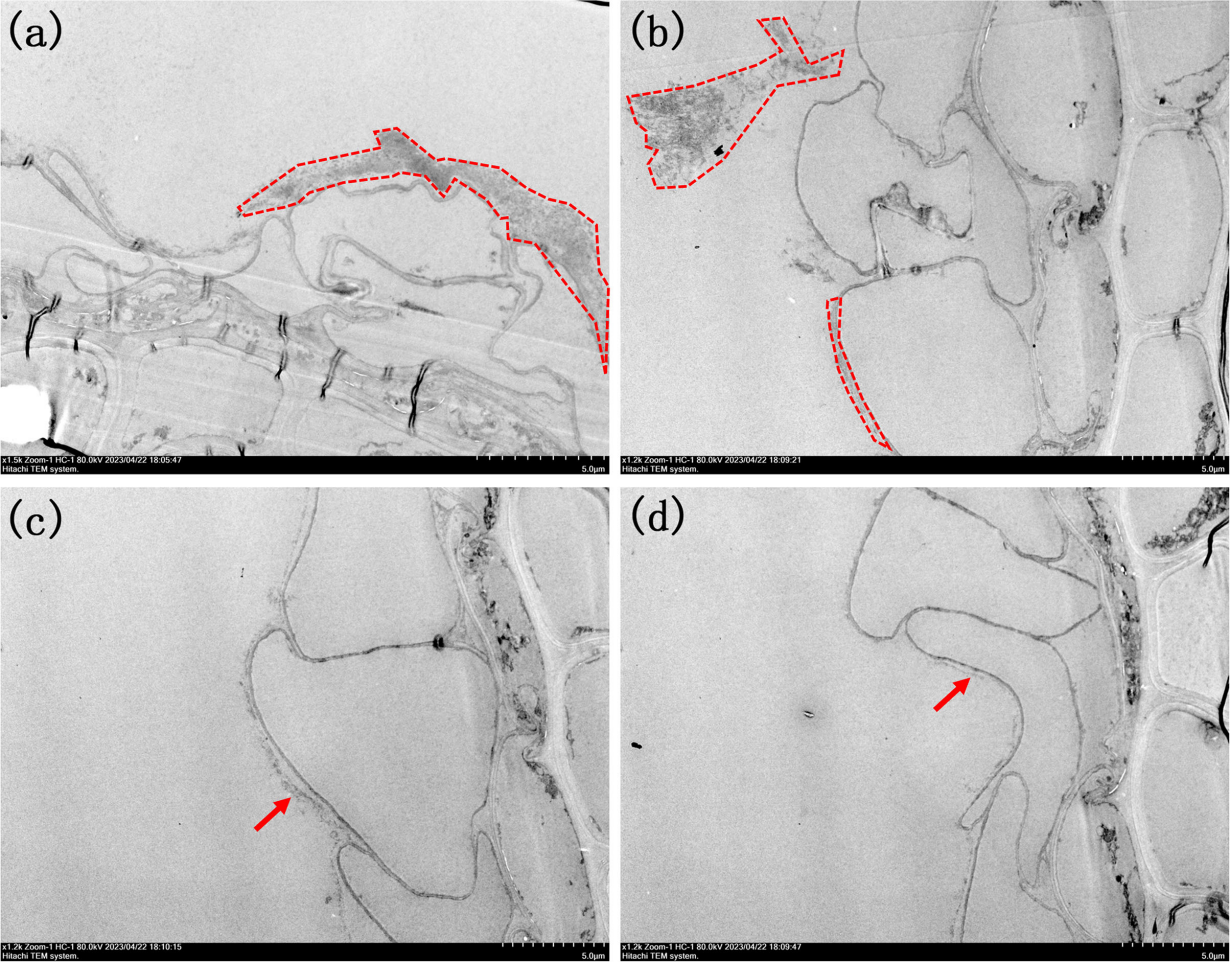
Morphologies of root TEM sections under different PO_4_^3-^ treatments. **(A)** 1500x by P0.01; **(B)** 1200x of P0.02; **(C)** 1200x of P0.033; **(D)** 1200xof P0.1. The iron plaques were outlined in image **(A, B)**. Arrows in image **(C, D)** highlight the presence of ultra-thin iron plaque.

To enhance the observation of iron surface responses to variations in the Fe^2+^ and PO_4_^3-^ concentrations within the nutrient solution, HRTEM was employed for examination after preparing ultrathin sections of the roots (**Figures 5**, **6**). When the Fe^2+^ concentration in the nutrient solution reached 0.4 mmol/L, a colloidal iron plaque with a thickness of approximately 10 μm was observed on the root surface, featuring tiny particles measuring approximately 200 μm at its center, as shown in **Figure 5A**. As the concentration of Fe^2+^ was further increased to 1.2 mmol/L, a surface layer with a thickness of only 2 μm consisting of aggregated small particles covered the root surface (**Figure 5B**). Upon closer examination, it was revealed that this layer consisted of nanoparticles ranging from one to several tens of particles, each measuring approximately 200 nm in diameter (**Figure 5C**). When the concentration of Fe^2+^ reached 2.0 mmol/L, the thickness of the particle layer increased to approximately 5 μm (**Figure 5D**). Finally, upon reaching a concentration of 3.2 mmol/L, there were no further increases in the thickness of the particle layer (**Figure 5E**). At this juncture, a distinct 3 μm and block-like hematite morphology emerged within the central region of the layer, which was enveloped by a colloidal coating (**Figure 5F**).

When maintaining a constant Fe^2+^ concentration and adjusting only the PO_4_^3-^ concentration in the nutrient solution, the results were consistent with the SEM observations (**Figure 6**). Upon addition of PO_4_^3-^ at a concentration of 0.01 mmol/L, a surface layer composed of small particles and with a thickness of 3 μm formed, which resembled the morphology observed upon adding Fe^2+^ at a concentration of 1.2 mmol/L (**Figure 6A**). However, when the concentration of PO_4_^3-^ in the nutrient solution was further increased to 0.02 mmol/L, it was observed that the thickness of this layer decreased to approximately 500 nm (**Supplementary Figures 3**, **4**). Additionally, small particle aggregates emerged on the outer sides of the root surfaces. This observation suggested that a decrease in the PO_4_^3-^ concentration made the iron plaque on the root surface more susceptible to detachment, as shown in **Figure 6B**. When the concentration of PO_4_^3-^ reached 0.033 mmol/L, there is a monolayer consisting of nanoparticles with a diameter of approximately 200 nm was observed on the iron plaque located on the root surface (**Figure 6C**). Upon reaching the maximum concentration of 0.1 mmol/L, the iron plaque was transformed into a colloidal form with a thickness less than 100 nm, predominantly composed of iron oxide (**Figure 6D**).

## 4 Discussion

In summary, the crystallization of iron plaque on rice roots began with the formation of a colloidal amorphous layer composed of ferrihydrite. As the rice grew and enhanced oxygen secretion throughout the growth cycle, colloidal ferrihydrite gradually underwent oxidation and crystallization, resulting in the development of a relatively weakly crystalline layer consisting of iron minerals that enveloped the root surface. Furthermore, as the oxygen secretion capability of the roots intensified, a fraction of the colloidal ferrihydrite was directly oxidized into more stable hematite particles.

The formation of iron plaques is not limited to the root surfaces of rice but also occurs in various aquatic plants, enabling them to adapt to flooding or other environmental stressors (Yang et al., 2021; Gu et al., 2022). The formation of iron plaques on plant root surfaces is significantly influenced by the concentrations of Fe^2+^ and PO_4_^3-^ in the root environment, as indicated by previous studies. Pi et al. proposed that the formation of iron plaques on roots was correlated with root growth, including the main root length, total root count, root surface area, and root volume (Pi et al., 2010). However, in this study, the quantity of iron plaques on the root surfaces were increased with the addition of Fe^2+^ in the enriched nutrient solution along with various indicators of root growth. Conversely, in the PO_4_^3–^enriched nutrient solution treatment, there was between iron plaque formation on the root surface and indicators of root growth. This resulted because the Fe^2+^ content promoted rice growth by significantly enhancing the root vitality while simultaneously oxidizing Fe^2+^ on the root surface, leading to an increase in iron plaque formation as the Fe^2+^ content increased. In contrast, there was a distinct variation in elevation with low PO_4_^3-^ treatments, which induced a state of low-phosphorus stress in the rice plants. This stress enhanced root vitality and increased the activity levels of pivotal enzymes such as SOD, POD, and CAT. Additionally, prior research showed that H_2_O_2_ was the prevalent oxidative substance and signaling molecule secreted by rice roots in response to adverse stress conditions. Consequently, the imposition of low-level phosphorus stress may enhance the exudation of H_2_O_2_ on the root surface, thereby facilitating the uptake and oxidation of Fe^2+^ ions and extensive formation of the iron plaque (Huang et al., 2022). In contrast, previous studies were specifically focused on the role of PO_4_^3-^ in the formation of iron oxides and demonstrated its inhibition of crystallinity, assembly processes, and transformations (Ustunol et al., 2021). However, increases in the PO_4_^3-^ concentration in the nutrient solution enhanced the growth environment for rice and promoted root development. Consequently, added PO_4_^3-^ resulted in increased indicators of rice root growth while reducing the iron plaque content.

The presence of iron plaque in rice roots increased the resistance of the rice plants to environmental stresses caused by heavy metals such as Cd and Pb, and this effect was closely related to the structural morphology of the iron plaque (Yan et al., 2021). Previous studies demonstrated that ferrihydrite has a greater capacity for heavy metal enrichment than hematite (Song et al., 2021). The presence of PO_4_^3-^ as an inhibitor suppressed further crystallization and oxidation of ferrihydrite, leading to the formation of a weakly crystalline or colloidal state, thereby inhibiting the formation of needle-like goethite or hematite (Banfield et al., 2000; Qafoku et al., 2020; Sheng et al., 2021). This was consistent with the phenomena observed in this study, where an increase in the PO_4_^3-^ level inhibited the transformations of weakly crystalline ferrihydrite small particles on the root surface into bulk hematite. Previous studies have shown that Fe^2+^ promoted the transformation of ferrihydrite into needle-like fibrous-like goethite, lepidocrocite (γ-FeOOH) and a small amount of hematite through electron transfer processes (Ustunol et al., 2021). In our study, we observed bulk hematite formation when the concentration of Fe^2+^ reached 3.2 mmol/L, but no needle-like particles were found. The appearance of hematite was attributed to both the addition of a large amount of Fe^2+^, which enhanced the rice root activity and oxygen-releasing capacity and facilitated rapid oxidation into relatively stable hematite particles, and to a dissolution-recrystallization process within the colloid layer on the outer surface of the hematite. When the Fe^2+^ concentration was only 0.4 mmol/L, the insufficient activity of the rice roots and weaker oxygen-releasing capacity resulted in crystallization within the colloid layer, leading to the formation of less stable ferrihydrite nanoparticles.

Additionally, it is noteworthy that the differences in iron plaque formation included the structure, morphology and thickness and also in the location. When treated with Fe^2+^, the iron plaques were in close proximity to the surface of the root cells. However, as depicted in **Figure 6**, a distinct gap exists between the root surface iron plaque and the root cells when exposed to different concentrations of PO_4_^3-^. This difference was attributed to the influence of PO_4_^3-^ on root activity, which enhanced the release distance of oxidative substances such as H_2_O_2_ or free radicals. Consequently, Fe^2+^ underwent oxidation to form an iron plaque even before coming into immediate contact with the root surface. These iron plaques served as effective barriers against harmful substances such as heavy metals located with a certain distance from the cells; however, they are susceptible to detachment from the root surface.

## Conclusion

Elevated Fe^2+^ concentrations in the nutrient solution resulted in improved indicators of rice root growth, as evidenced by an increase in DCB iron content, enhanced root vitality, and a notable surge in the activities of the SOD, POD, and CAT enzymes. Conversely, increasing the PO_4_^3-^ concentration led to various markers of increased root growth. However, with low-level phosphorus stress, higher PO_4_^3-^ concentrations resulted in decreased DCB iron content and root vitality. The trends of the SOD, POD, and CAT enzyme activities exhibited initial rises followed by subsequent declines. The amorphous iron oxide minerals on the root surface gradually transformed into ferrihydrite particles measuring approximately 5 μm due to an increase in the Fe^2+^ concentration. In contrast, a higher PO_4_^3-^ concentration was correlated with a reduction in the quantity of iron plaques on the root surface. Furthermore, the thickness of the granular ferrihydrite-based iron plaque regressed from 3 μm to a monolayer composed of a particle-based film. Importantly, the PO_4_^3-^ treatment enlarged the distance between the root surface cells and the iron plaque. The intricate mineral morphologies of these plaques proved sensitive to oxygen exudation from the roots. The initial phase involved the formation of amorphous iron oxide films, which eventually dissolved and crystallized into ferrihydrite particles when the oxidation capacity increased along with excessive Fe^2+^ concentrations, resulting in decreased plaque formation. Therefore, it is crucial to pay meticulous attention to shifts in the crystal structure, morphology and reactivity when evaluating root surface iron plaques.

## Data availability statement

The original contributions presented in the study are included in the article/**Supplementary Material**. Further inquiries can be directed to the corresponding author.

## Author contributions

HH: Formal analysis, Methodology, Validation, Writing – original draft. LB: Methodology, Validation, Writing – review & editing. LW: Data curation, Formal analysis, Writing – review & editing. FZ: Supervision, Writing – review & editing. XL: Project administration, Resources, Writing – original draft, Writing – review & editing. LQ: Formal analysis, Methodology, Supervision, Writing – original draft. YL: Supervision, Writing – review & editing.
