# Supplementary material for: The effects of different iron and phosphorus treatments on the formation and morphology of iron plaque in rice roots (Oryza sativa L)
Source: Front Plant Sci. 2024 Jan 8;14:1304505. doi: 10.3389/fpls.2023.1304505 (PMC10800492; doi:10.3389/fpls.2023.1304505)
Supplement: Supplementary file 1 [file DataSheet_1.docx]

**Supporting Information**

**The Effects of Different Iron and Phosphorus Treatments on the Formation and Microscopic Morphology of Iron Plaque in Rice Roots (*Oryza sativa L*)**

Haoran Hu^1^, Liyan Bi^1^, Lei Wang^1^, Fangdong Zhan^1^, Xinran Liang^1^*, Li Qin^1^, Yuan Li^1^

1. *College of Resources and Environment, Yunnan Agricultural University, Kunming 650201, China*


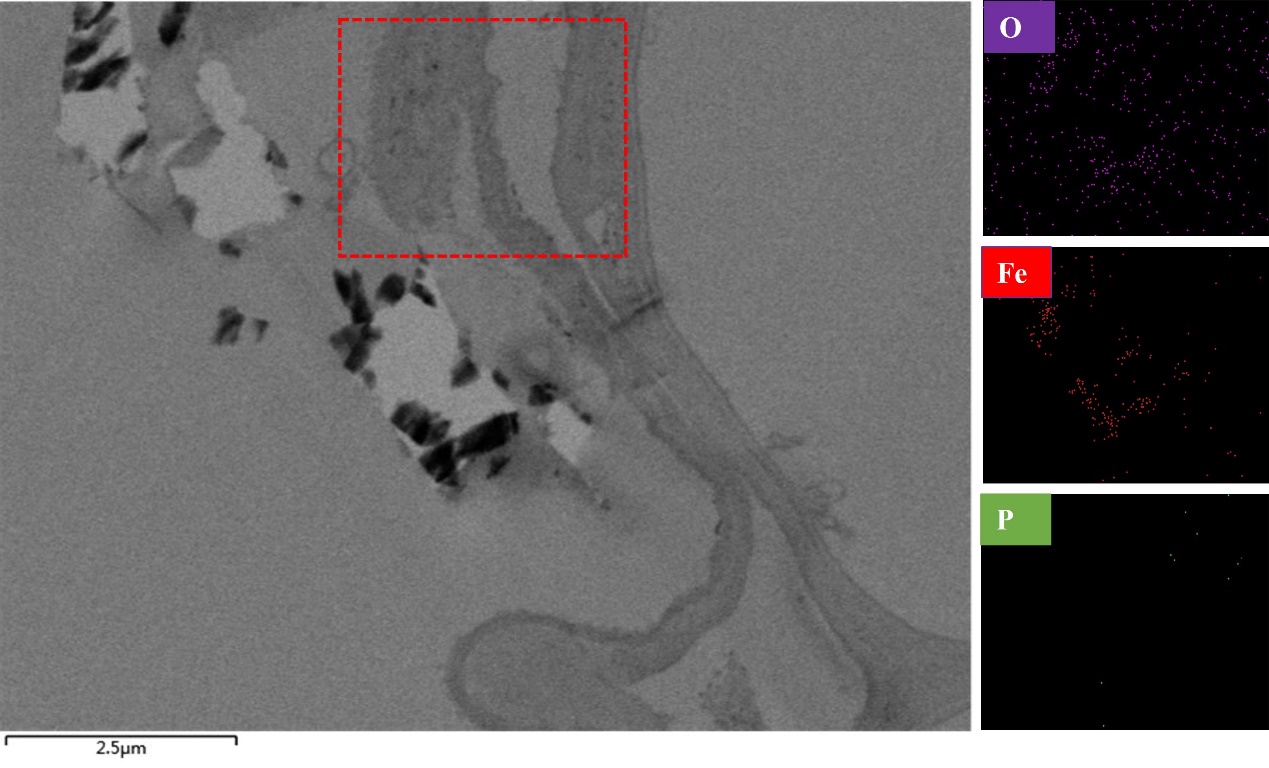
Figure S1 Morphologies of root TEM sections under P0.02treatments. On the right is the elements distribution mapping of iron plaque in the red rectangular area.


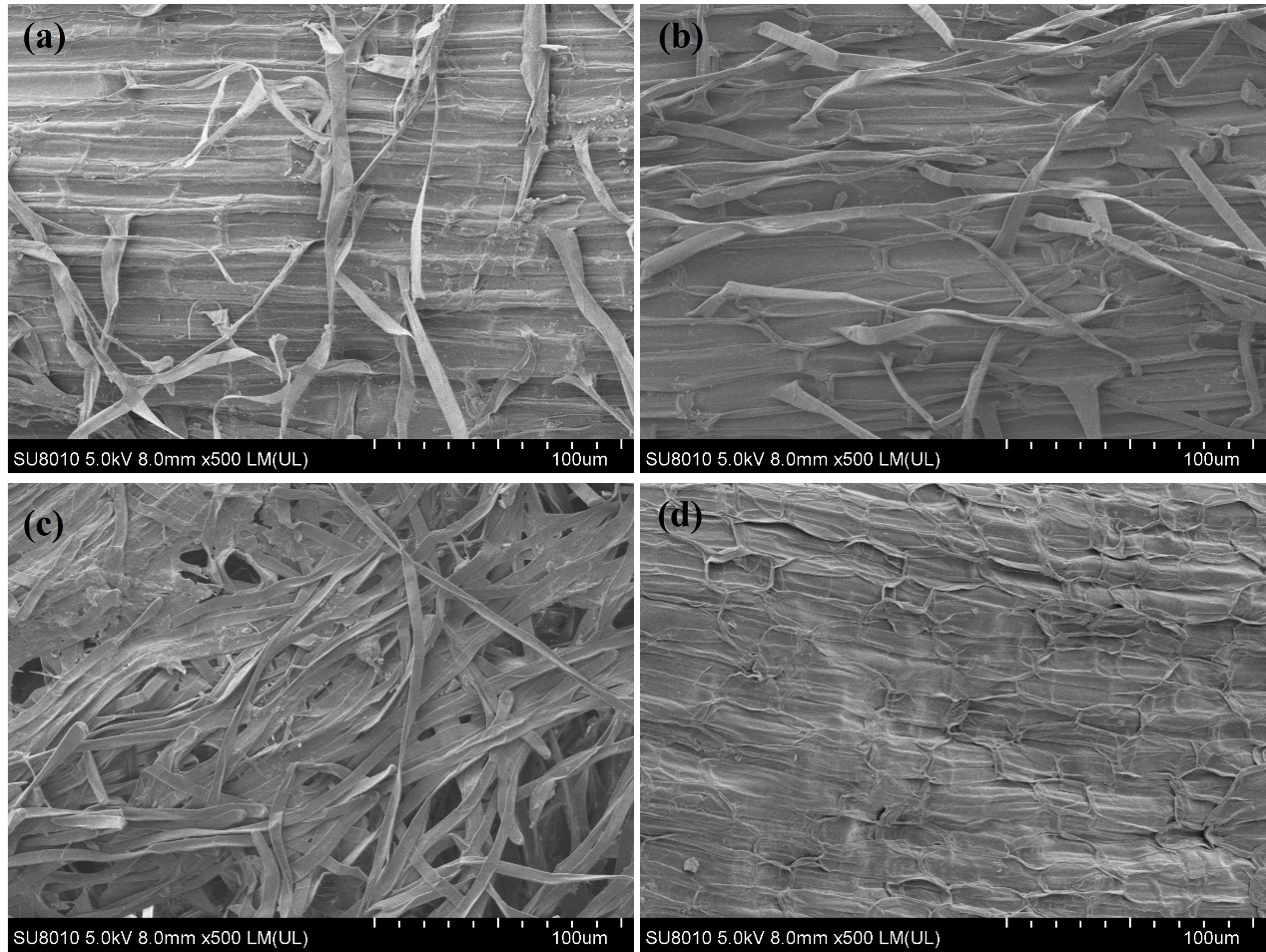
Figure S2. Morphologies of root 500x SEM sections under with different Fe^2+^ treatments, (a) Fe0.4; (b) Fe1.2; (c) Fe2.0; (d) Fe3.2

**
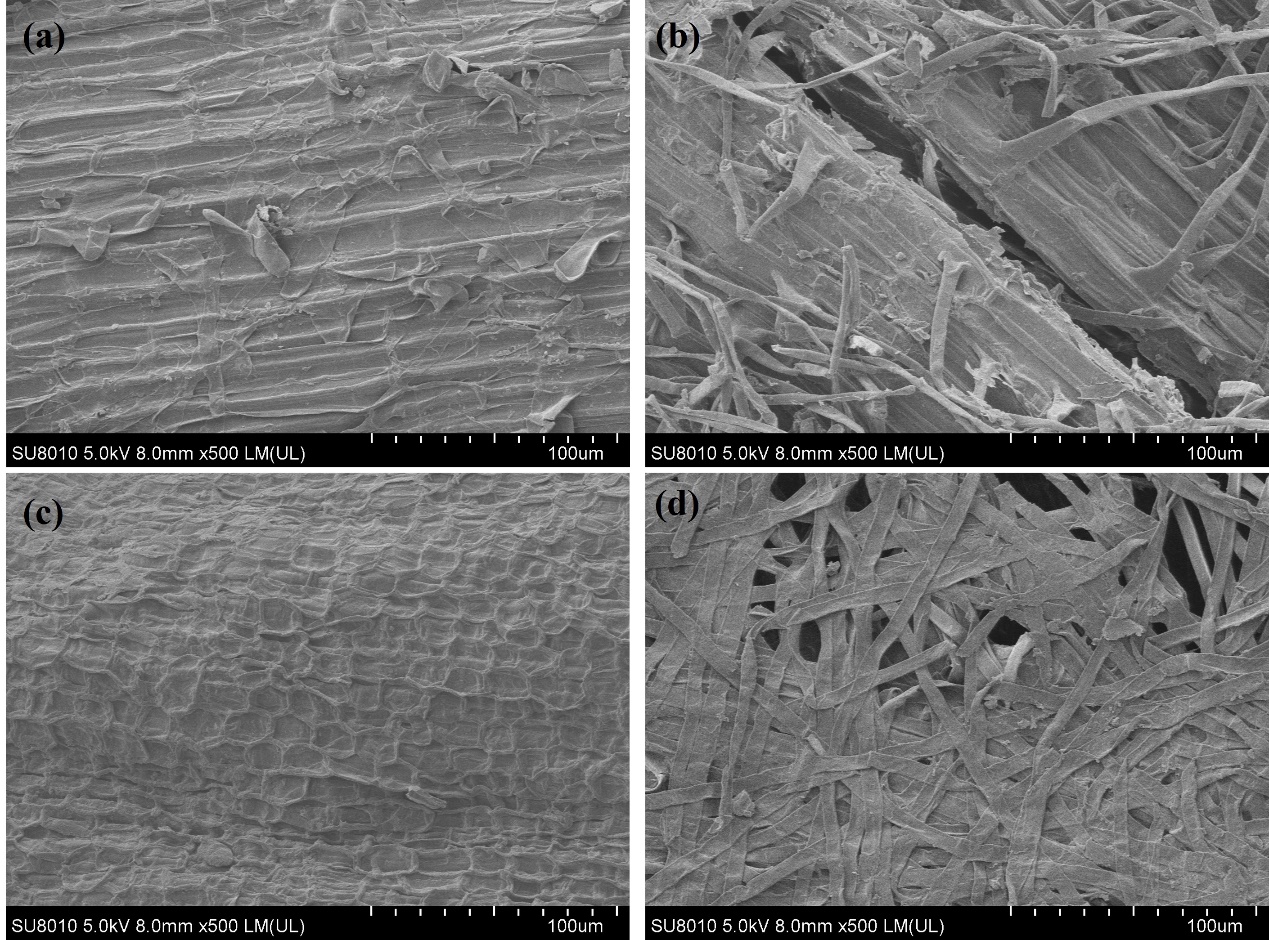
**

Figure S3. Morphologies of root 500x SEM sections under with different PO_4_^3-^ treatments, (a) P0.01; (b) P0.02; (c) P0.033; (d) P0.1

**
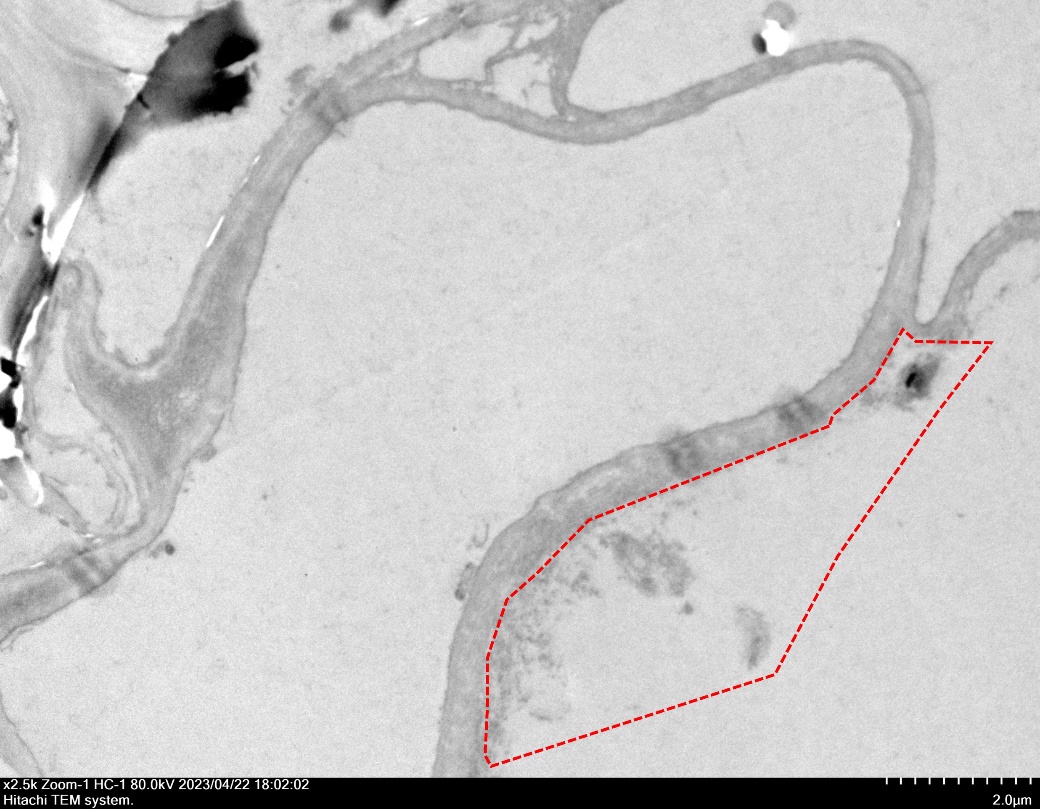
**Figure S4. Morphologies of root TEM sections under different PO_4_^3-^ treatments. 1500x of P0.02. The iron plaques were outlined in image

**
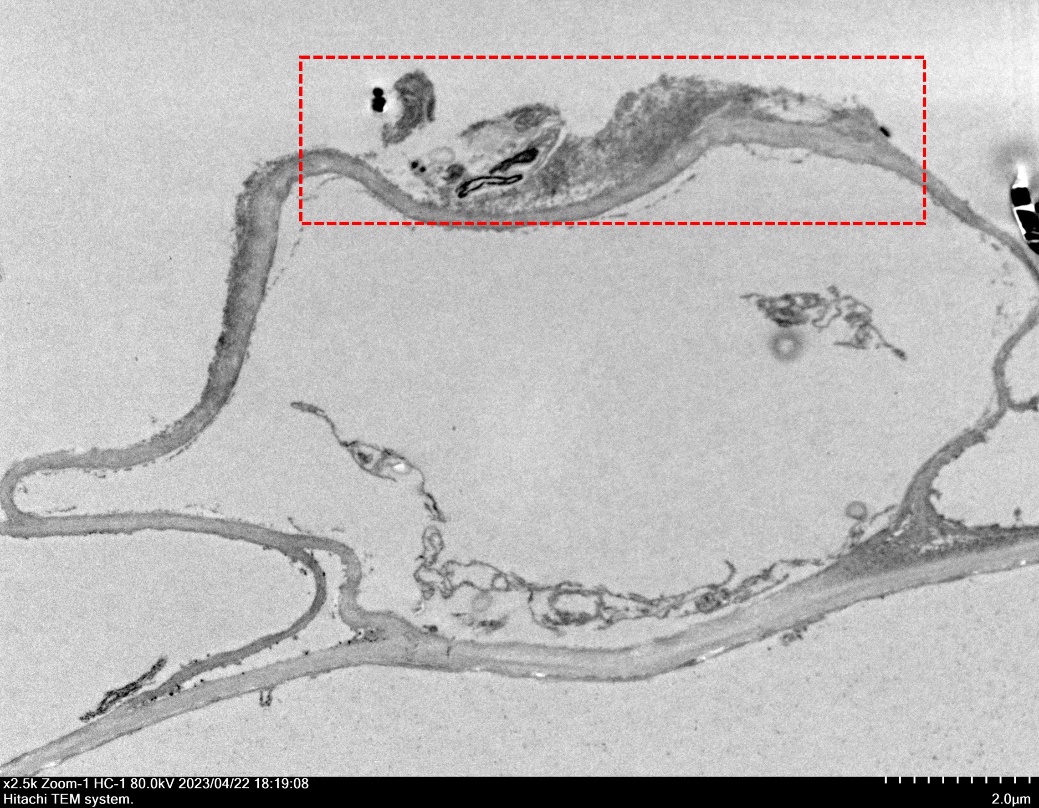
**Figure S5. Morphologies of root TEM sections under different Fe2+ treatments. 2500x of Fe3.2. The iron plaques were outlined in image
